# Electromagnetic tomographic cerebral angiography

**DOI:** 10.1038/s41598-024-51632-4

**Published:** 2024-01-20

**Authors:** Serguei Semenov

**Affiliations:** EMTensor GmbH, 1220 Vienna, Austria

**Keywords:** Biophysics, Cardiology, Engineering, Physics

## Abstract

World Health Organization stated that “Cardiovascular diseases (CVDs) are the leading cause of death globally. Angiography is an important method in diagnostic of CVD. Standard-of-Care methods of angiography, such as X-Ray or CT- or MRI- angiography methods, being accurate and widely adopted in clinical practice, are bulky, expensive and energy in-efficient. X-ray and CT- angiography methods are also potentially hazardous as techniques require the use of ionizing contrast agents. Electromagnetic tomography (EMT) is an emerging medical imaging modality. EMT is applicable for safe functional imaging but suffers from a limited spatial resolution because of relatively large wavelength of electromagnetic radiation as compared to sizes of biological targets of particular interest, such as, for example blood vessels. Novel approach and method, presented in the study is capable to overcome such limitations and provide a mean for a dynamic, on-line EMT angiography. New method of EMT angiography was presented in application to cerebral angiography. Achieved imaging results clearly demonstrate applicability of the method for detecting small cerebral vessels of the diameter as small as 1.3 mm and to distinguish vessels with different dimensions. The technical challenges in the development of angiography capable EMT systems are assessed and discussed.

## Introduction

World Health Organization stated that “Cardiovascular diseases (CVDs) are the leading cause of death globally, taking an estimated 17.9 million lives each year. CVDs are a group of disorders of the heart and blood vessels and include coronary heart disease, cerebrovascular disease, rheumatic heart disease and other conditions”^1^. It also states that most CVDs can be prevented and early diagnostic and treatment are critical. A stroke or cardiovascular accident is the event of acute compromising of perfusion of brain tissues, is the fifth cause of death in the USA and is the leading cause of disability in the USA^2^.

Electromagnetic tomography (EMT) is an emerging medical imaging modality which utilizes an electromagnetic radiation from a non-ionizing portion of the electromagnetic spectrum (for example, in a frequency range of about 0.01 GHz to about 10 GHz) for interrogation of biological object under the study. In this portion of electromagnetic spectrum, tissues are differentiated and, consequentially, can be imaged based on the differences in their dielectric properties. It is known that dielectric properties of tissues with high (such as muscle) and low (such as fat and bone) water content are significantly different^3,4^. During the last decades the changes in dielectric properties of tissues caused by various physiological and pathological alterations, such as blood content, ischemia, infarction, hypoxia and malignancy have been intensively studied. In our pre-clinical studies, we’ve shown that dielectric properties of brain soft tissues and skeletal muscles tissues are sensitive to the blood content of tissues^5–7^ and dielectric properties of myocardium are sensitive to its blood content and hypoxia with almost immediate effect following intervention^8–10^. An additional importance of those findings is that spectral changes in dielectric properties of tissues caused by acute blood deficiency and acute hypoxia are different. Another importance of those findings is that the changes in dielectric properties of tissues have timely dependence allowing for an assessment of timely development of tissue viability/damage and/or an assessment of an efficacy of treatment. Those findings present a strong scientific foundation for the development of novel, clinically viable imaging technology.

“Angiography or arteriography is a medical imaging technique used to visualize the inside, or lumen, of blood vessels and organs of the body, with particular interest in the arteries, veins, and the heart chambers. Modern angiography is performed by injecting a radio-opaque contrast agent into the blood vessel and imaging using X-ray based techniques such as fluoroscopy”^11^. Standard-of-Care methods of angiography, such as X-Ray or CT- or MRI- angiography methods, being accurate and widely adopted in clinical practice, are bulky, expensive and energy in-efficient. X-Ray and CT- angiography methods are also potentially hazardous as techniques require the use of ionizing contrast agents. Additionally, those standard-of-care methods of angiography are unable to provide on-line, safe, cost and energy efficient assessment of both tissue viability and status of vessels especially in mobile, bed-site settings. This data might be of critical importance for example, during medical emergencies, at nursing homes, during anesthesia, surgery or childbirth, to name a few. Therefore, there is a need for a technology that is capable of addressing such issues of critical medical importance in mobile, bed-site settings within on-line, safe, cost- and energy-efficient fashion. The data acquisition process of modern EMT scanners is electronically controlled and is fast. One frame of full tomographic data can be acquired within msec timing, allowing, for example, for circulation gated imaging with up to 100 acquired data frames per cardiac cycle. EMT technology is completely safe. It uses electromagnetic radiation from non-ionizing portion of the spectrum at power level lesser than power level used in mobile phones.

Electromagnetic tomography is applicable to functional imaging of biological objects^7,12–14^ but suffers from a limited spatial resolution because of relatively large wavelength of electromagnetic radiation as compared to sizes of biological targets of particular interest, such as, for example blood vessels. For example, a wavelength of electromagnetic radiation at a typical frequency of 1GHz, used for cerebral imaging is about 4.7cm within a brain tissue, which is significantly larger than the dimensions of even large cerebral vessels. However, novel approach and method, presented here is capable to overcome such limitations and provide a mean for a dynamic, on-line electromagnetic tomographic angiography.

Methods of images reconstruction in EMT are mathematically complex and computationally extensive. Electromagnetic diffraction and interference phenomenon, being almost negligible in convenient methods of biomedical tomography (such as CT), turn into a major factor required special attention. In general case, it will lead to an iterative process with: i) the necessity of finding the solution of complicated vector Maxwell equations within an imaging domain from all sources of EM radiation used at each iteration and to ii) data inversion at each iteration step. Different methods of EMT images reconstructions have been developed, tested and successfully applied for imaging of biological objects, including the most recently developed AI based methods.

The EMT imaging of brain, being the task of significant clinical importance, presents a very complicated, high dielectric contrast problem. The challenge is to reconstruct hidden properties of deep brain tissues effectively shielded by a high dielectric contrast shield, comprising the skull with low dielectric permittivity and cerebrospinal fluid with high dielectric permittivity: from 12 to 60 correspondingly at GHz region of EM spectrum. The challenging problem attracts serious attentions of scientific community^13–22^.

The novel EMT angiography method presented in this study is illustrated in an application for cerebral angiography. However, it can be applicable for other fields of angiography, for example to a coronary angiography.

The number of publications in the field of electromagnetic (microwave) angiography is limited. To the best of our knowledge there are publications from group of Dr Lauteslager with colleagues focusing on exploration of ultrawide band technology using radar-on-chip devices for microwave imaging of cardiovascular system^23^ and feasibility assessment of ultrawide band technology for monitoring of arteries using phantom system^24^. To the best of our knowledge there are no publications covering the subject of electromagnetic tomographic (EMT) angiography and specifically EMT cerebral angiography.

The paper organized as: following the Introduction section, the method of Electromagnetic Tomographic Angiography is described in Methods section, together with two virtual human head models. Results of the study are presented and discussed in the Results and Discussion section, followed by Conclusion remarks.

## Methods

### Electromagnetic tomographic angiography

**Figure 1:**
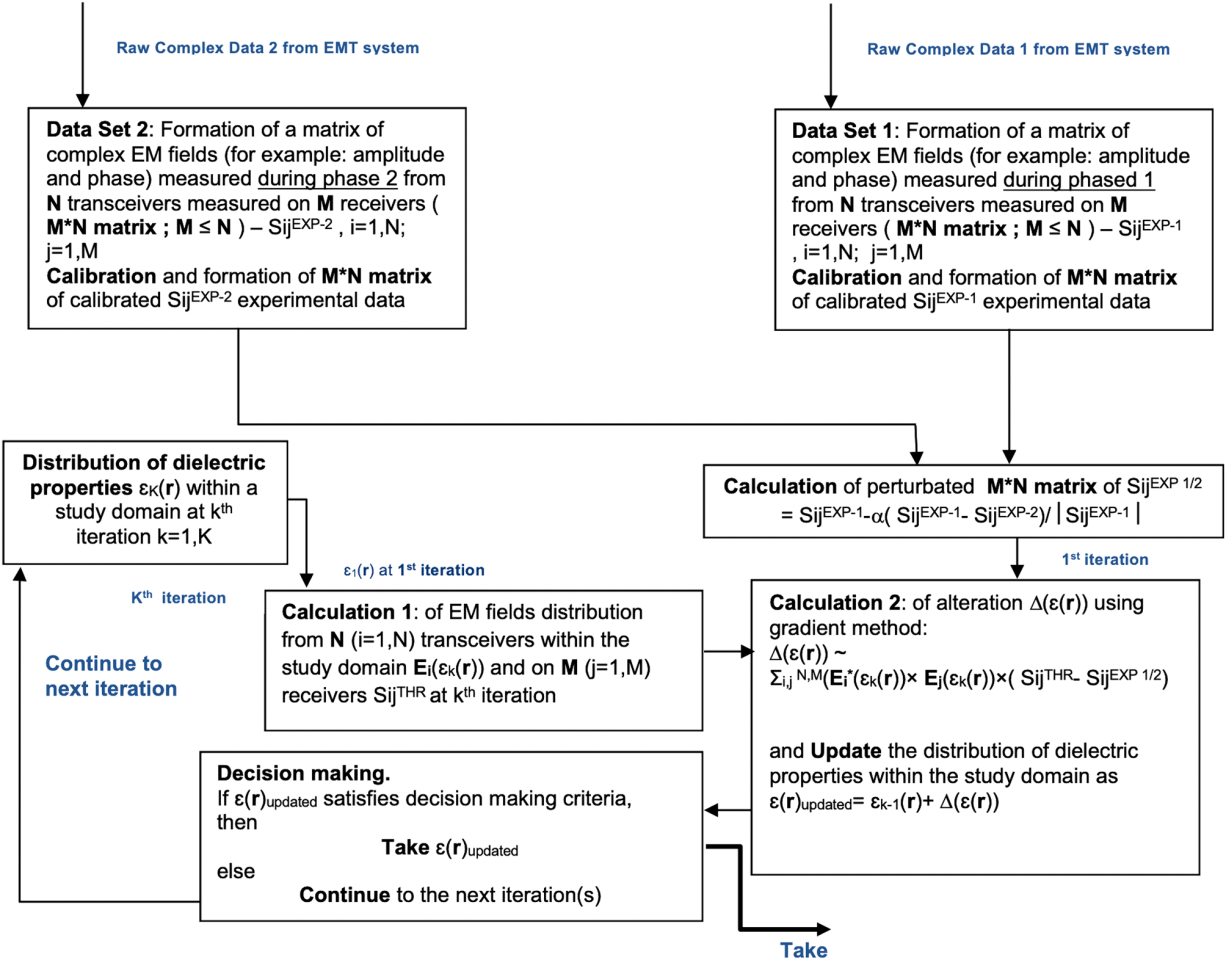
Flow-chat of the algorithm of the method of Electromagnetic Tomographic Angiography.

**Figure 2:**
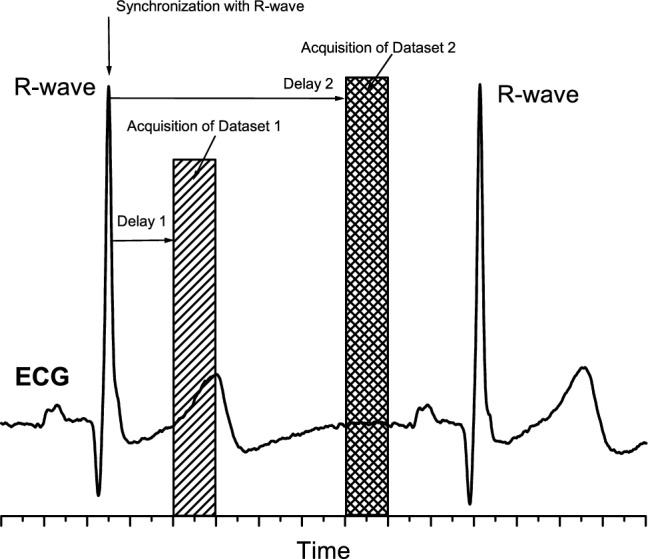
Schematic illustration of synchronization of EMT data acquisition with circulation and acquisition of two EMT datasets of raw complex EM data.

The problem of Electromagnetic Tomographic Angiography (EMTA) is formulated as following. The object with unknow three-dimensional (3D) distribution of dielectric properties ε(***r***) is positioned within an imaging domain Φ(***r***). In general case, *N* transmitting antennas and *M* receiving antennas are placed on the boundary of an imaging domain and/or within an imaging domain but outside an object of interest (object domain). In specific case of transceivers, when all antennas and antenna’ channels have both transmitting and receiving capabilities, the total number of antennas is equal *N*. The flow-chat of the algorithm of EMTA method is presented in Fig. 1. The acquisition of raw complex tomographic data within EMTA system is synchronized with circulation as illustrated in Fig. 2. In this example the synchronization is performed with R-wave of ECG. Two independent datasets of complex data (for example, real and imaginary parts or amplitude and phase) are acquired during each cardiac cycle as also illustrated in Fig. 2. Each dataset presents a matrix of complex EM fields from *N* transmitters measured on *M* receivers—so-called *S*_*ij*_^*EXP-1*^ and *S*_*ij*_^*EXP-2*^ matrixes for dataset 1 and dataset 2 correspondingly. Each dataset is independently calibrated. The dielectric properties of so-called matching media, which is the media occupying the whole imaging domain except an object of the study domain, is considered to be known or independently measured, isotropic within the whole domain and equal to ε_0_ (***r***). Therefore, having: i) measured at least two matrixes *S*_*ij*_^*EXP-1*^ and *S*_*ij*_^*EXP-2*^ of complex electromagnetic fields on the boundary of an imaging domain (or within an imaging domain but outside an object domain) and ii) known or measured dielectric properties of matching media ε_0_ (***r***), the goal is to reconstruct 3D distribution of dielectric properties ε(***r***) within an object domain, which is an image of the object under the study.

The problem is formulated as minimization problem as to find a minimum of functional $M\left(\upvarepsilon \left({\varvec{r}}\right)\right)$ (Eq. (1) below), such that an obtained distribution of ε(***r***) within on object domain minimizes the norm between measured *S*_*ij*_^*EXP*^ and theoretically calculated *S*_*ij*_^*THR*^. Regularization $\Omega \left(\upvarepsilon \left({\varvec{r}}\right)\right)$ with regularization parameter γ might be added to stabilize a solution.

1 $$M\left(\upvarepsilon \left({\varvec{r}}\right)\right)={\sum_{i,j}^{N,M}\left|\left|{S}_{ij}^{EXP}- {S}_{ij}^{THR}\right|\right|}^{2}+\upgamma *\Omega \left(\upvarepsilon \left({\varvec{r}}\right)\right)$$

Within EMTA method, the matrix *S*_*ij*_^*EXP*^ is substituted by so-called perturbated matrix *S*_*ij*_^*EXP-EMTA*^. The calculation of perturbated *S*_*ij*_^*EXP-EMTA*^ matrix is performed as following:

2 $${S}_{ij}^{EXP-EMTA}={S}_{ij}^{EXP-1}+ \mathrm{\alpha }\frac{\left({S}_{ij}^{EXP-1}- {S}_{ij}^{EXP-2}\right)}{\left|{S}_{ij}^{EXP-1}\right|}$$

where |Sij^EXP-1^| is a norm of complex Sij^EXP-1^ and α—is a parameter chosen by a trial method. It is obvious that dataset indexes 1 and 2 can be exchanged. This is an important step separating EMT angiography from classical EMT, when non-perturbated *S*_*ij*_^*EXP*^ matrix is used. In cerebral application of EMTA method, the assumption is that a short time separating the acquisition of two different datasets within a single cardiac cycle allows for “a frozen” object approximation within a whole volume of brain except for circulation dependent portions, ie blood vessels. In other than cerebral application of EMTA, for example, in EMTA of coronary vessels, this approximation is invalid in general case as there is a need to account for movements caused by electro-mechanical phenomenon, ie myocardial contraction. In regards to the strategy of choosing the value of parameter α. It is obvious that if α is at zero level, the matrix *S*_*ij*_^*EXP*^ is not perturbated and image reconstruction will be identical to classical, not perturbated case. On another site, if parameter α is high compared with a typical amplitude of components of *S*_*ij*_^*EXP*^ matrix, then iterative images reconstruction process will not converge. Therefore, the strategy of a trial method is to choose largest parameter α, so vessels are reconstructed while keeping a convergence of the iteration process.

For images reconstruction we employed iterative process and used gradient method to solve inverse problem at each iteration. In block “Calculation 1”, the direct problem is solved: the EM fields distribution is calculated within an imaging domain from N transmitters. The complex values of EM fields on M receivers are also calculated, forming a matrix of *S*_*ij*_^*THR*^ at each iteration. At each iteration (for example at kth iteration) the distribution of dielectric properties within an imaging domain (including an object under the study) ε_k_(***r***) is either taken as updated value from k-1 iteration as ε_k_(***r***) = ε_k-1_(***r***) + β_k_*Δ( ε(***r***)) or assumed to be known at the 1st iteration. We took homogeneous distribution of dielectric properties of known (measured independently) matching media ε_1_(***r***) = ε_0_ as an initial guess at the 1st iteration. β_k_—is a step coefficient with an initial value chosen by a trial method and updated during iteration process depending on the speed of the convergence. The alteration of dielectric properties Δ(ε(***r***)) (inverse problem) at kth iteration is calculated by a conjugate gradient method presented in details elsewhere^25–27^ in “Calculation 2” block as following:

3 $$\Delta \left( {\upvarepsilon }_{k}\left({\varvec{r}}\right)\right)={\sum }_{ij}^{N,M}({E}_{i}^{*}({\upvarepsilon }_{k}({\varvec{r}}))\times {E}_{j}({\upvarepsilon }_{k}({\varvec{r}})))\times ({S}_{ij}^{THR}-{S}_{ij}^{EXP-EMTA})$$

where: *E*_*i*_ and *E*_*j*_ are the EM field distributions from transmitting antenna *i (i* = *1 to N)* and from receiving antenna (simulating as in transmitting mode) *j (j* = *1 to M)* within an imaging domain respectfully; *—denotes complex conjugation.

The calculation usually runs until it reaches the conversion of at least 0.98 (Decision Making criteria in the Decision block). To avoid possible “inverse” crime we run model simulation on polar grids (128*96*63) (FRZ) while solve an inverse problem on cartesian grids 200*200*100 (XYZ). We also add random 5% noise independently to real and imaginary parts of raw simulated data prior its use in inverse problem and solve inverse problem part with lesser accuracy. 64 transmitting/receiving antennas were equidistantly position on the perimeter of a cylinder with radius 12 cm.

### Virtual human head model 1

**Figure 3:**
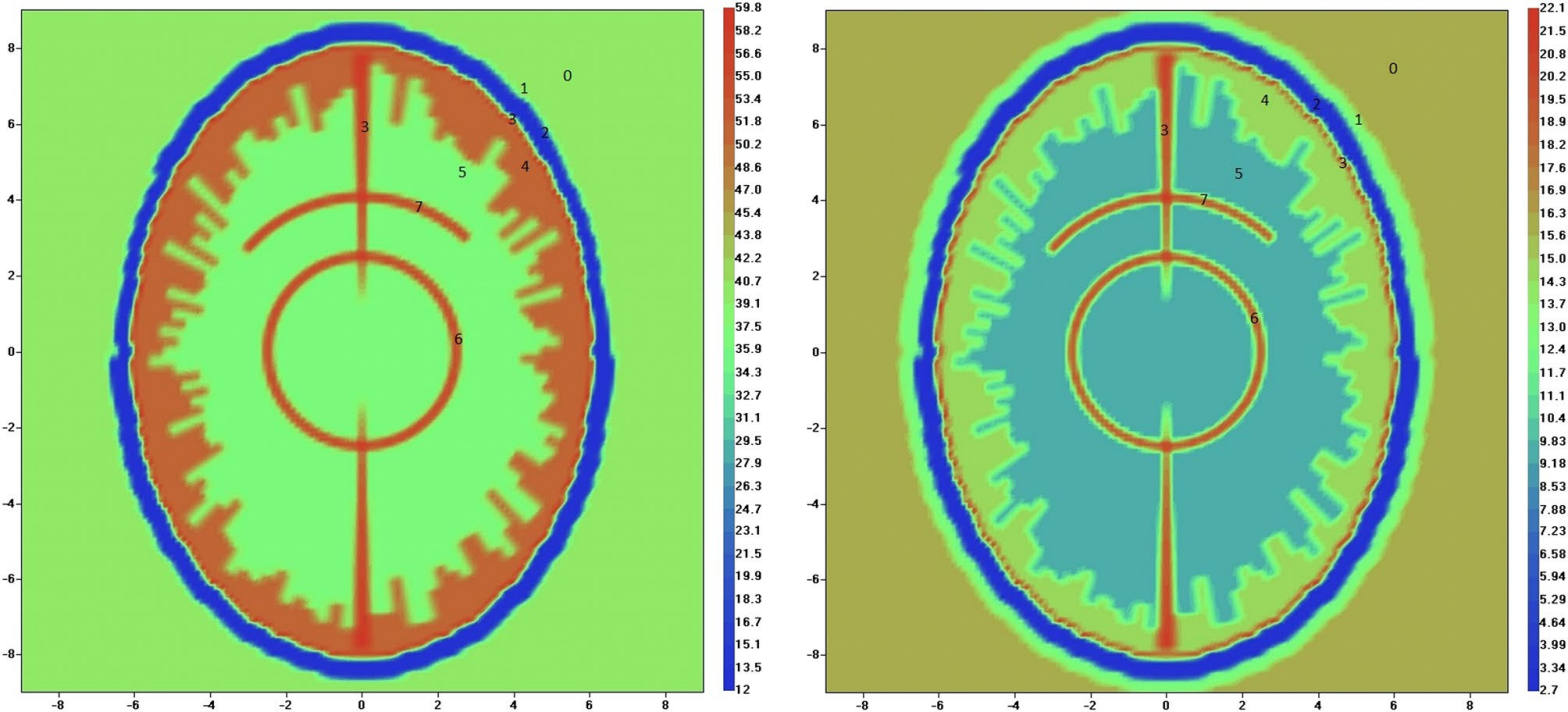
Virtual model of human head used in the study: left is real part and right is imaginary part of dielectric properties. Scales are in [cm]. Systolic phase is shown for virtual model 1. 0—matching media; 1. skin; 2. skull; 3. Cerebro-Spinal Fluid (CSF); 4. gray matter; 5. white matter; 6. circle of Willes; 7. blood vessels.

We used a 2D virtual model of human head as a cylindrical ellipsoid with main semi-axes of 7 [cm] and 9 [cm] positioned inside of cylindrical circular imaging domain with radius 12.5 cm and height 8 cm (Fig. 3). The overall dimensions of a human head model employed in the study are within published anthropometric values for adult human^28^. The dielectric properties of a media inside of an imaging domain was ε_0_ = 40 + j16 at frequency of 1.5 GHz used in the study. Transmitting/receiving antennas were located inside of an imaging domain at radius of 12 cm. The anatomic and dielectric parameters of the model is summarized in Table 1. The geometry of blood vessels’ parts of the model (circle of Willes and vessels) is inspired by the cerebroarterial statistical atlas^29^ obtained from 603 multi-center datasets (including the artery probability atlas and the mean artery radius atlas presented on Fig. 2 of the reference). To account for the differences between systolic and diastolic phases, blood vessels inclusions are filled with tabulated values of blood (Fig. 3 and Table 1) in systolic phase, while in diastolic phase the dielectric properties of vessel inclusions are assumed to be equal to the dielectric properties of white matter. Of course, this approach is a simplification and a bit of exaggeration of possible differences between systolic and diastolic phases. However, giving the larger wavelength of EM radiation as compared with the vessel dimension (47 mm vs 1.3 mm) and an absence of a reliable data for circulatory related variations in dielectric properties, we assumed that the approach is acceptable as an initial approximation in this proof of concept study. We extended this approach in model 2, presented below.

**Table 1:** Parameters of the virtual human models 1 and 2.

Tissue/media	Geometry (cm)	Re	Im
0. Matching media	Within all imaging domain except a head model	40	16
1. Skin	Thickness 0.5 except two pterion regions	39.4	12.8
2. Skull	Thickness 0.7 except two pterion regions 0.3	12.0	2.7
3. Cerebro-spinal fluid (CSF)	Thickness 0.2	59.9	22.2
4. Gray matter	Thickness 1.2 with random to white matter boundaries	50.7	14.7
5. White matter	Remaining brain volume with random to gray matter boundaries	37.5	9.5
Model 1			
Circle of Willes/systolic	Radius 2.5. Vessels dimensions: XY = 1.3 mm; Z = 3.8 mm	59.9	22.2
Blood vessels/systolic	Vessels dimensions: XY = 1.3 mm; Z = 3.8 mm	59.9	22.2
Circle of Willes/diastolic	Radius 2.5. Vessels dimensions: XY = 1.3 mm; Z = 3.8 mm	37.5	9.5
Blood vessels/diastolic	Vessels dimensions: XY = 1.3 mm; Z = 3.8 mm	37.5	9.5
Model 2			
Circle of Willes/systolic	Radius 2.5. Vessels dimensions: XY = 2.6 mm; Z = 3.8 mm	59.9	22.2
Blood vessels/systolic	Vessels dimensions: XY = 2.6 mm; Z = 3.8 mm	59.9	22.2
Circle of Willes/diastolic	Radius 2.5. Vessels dimensions: XY = 1.3 mm; Z = 3.8 mm	60.8	19.7
Blood vessels/diastolic	Vessels dimensions: XY = 1.3 mm; Z = 3.8 mm	60.8	19.7

The dielectric properties (Re—real part and Im—imaginary part) are presented for frequency 1.5 GHz used in the study.

### Virtual human head model 2

With this model we used the same anatomic-dielectric model as in model 1, except the particulars of the model related to systolic and diastolic phases of circulation. Two approximations have been made. Firstly, in order to simulate circulatory pulse wave, we doubled the vessel diameter in systolic phase versus diastolic phase, from 1.3 mm to 2.6 mm. We understand that this might be overestimated for future quantitative studies based on the data from MRI study^30^, but acceptable on this qualitative, proof of concept report. Secondly, the differences in dielectric properties of blood (Hb) at systolic (oxy) and diastolic (deoxy) phases was accessed as following. The availability of scientific reports on dielectric properties of oxyHb and deoxyHb is limited at present. We based our estimations on the publication from Latypova with colleagues^31^. They reported the dielectric properties of different concentrations of oxyHb and deoxyHb in Phosphate Buffered Saline (PBS) , (pH = 7.4 at 25C). Based on the data presented, we estimated that ε_diastolic_ = 1.015*ε’_systolic_ + j(0.886*ε’’_systolic_).

## Results and discussion

**Figure 4:**
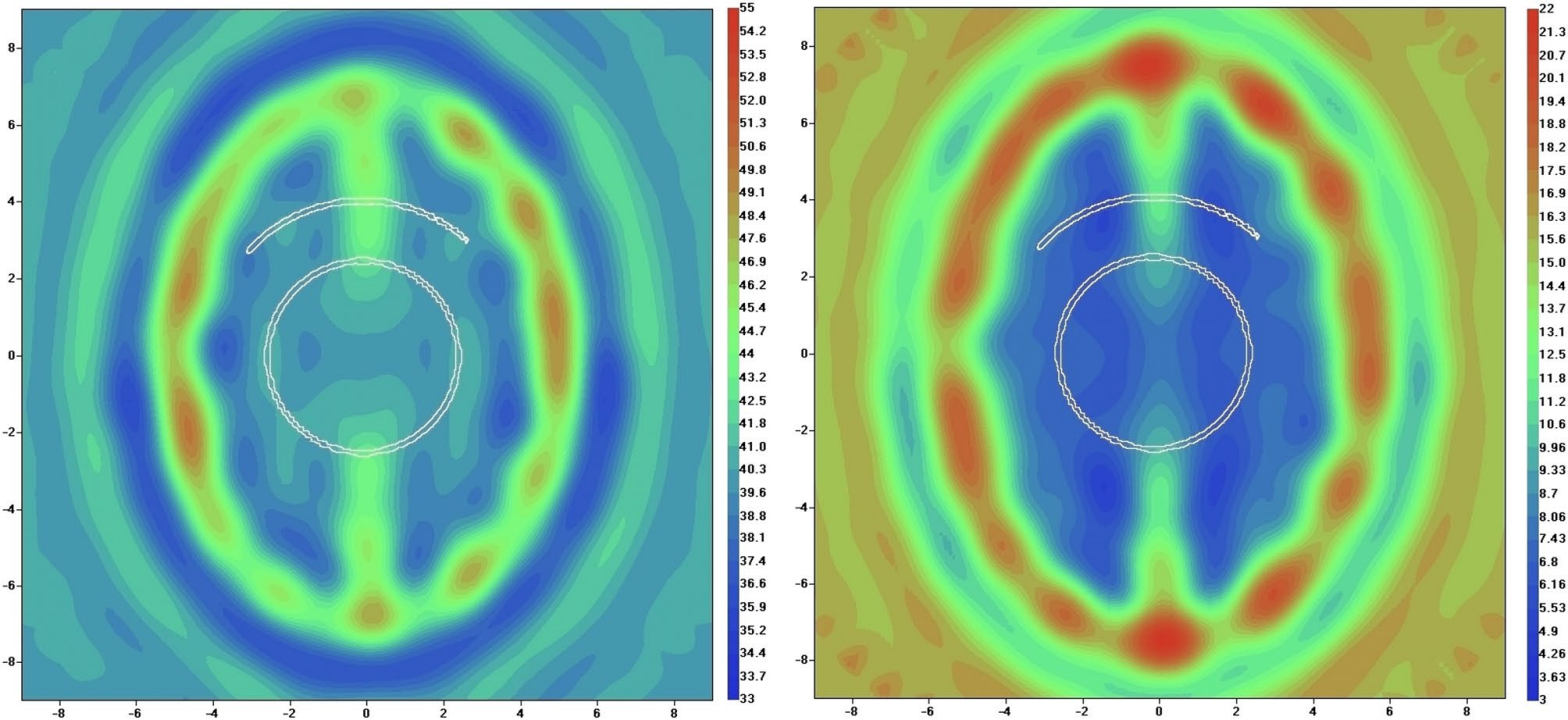
Reconstructed images of human head model 1 in systolic phase using EMT non-perturbated approach: left is real part and right is imaginary part of dielectric properties. Scales are in [cm]. The counter of vessel is white coloured.

**Figure 5:**
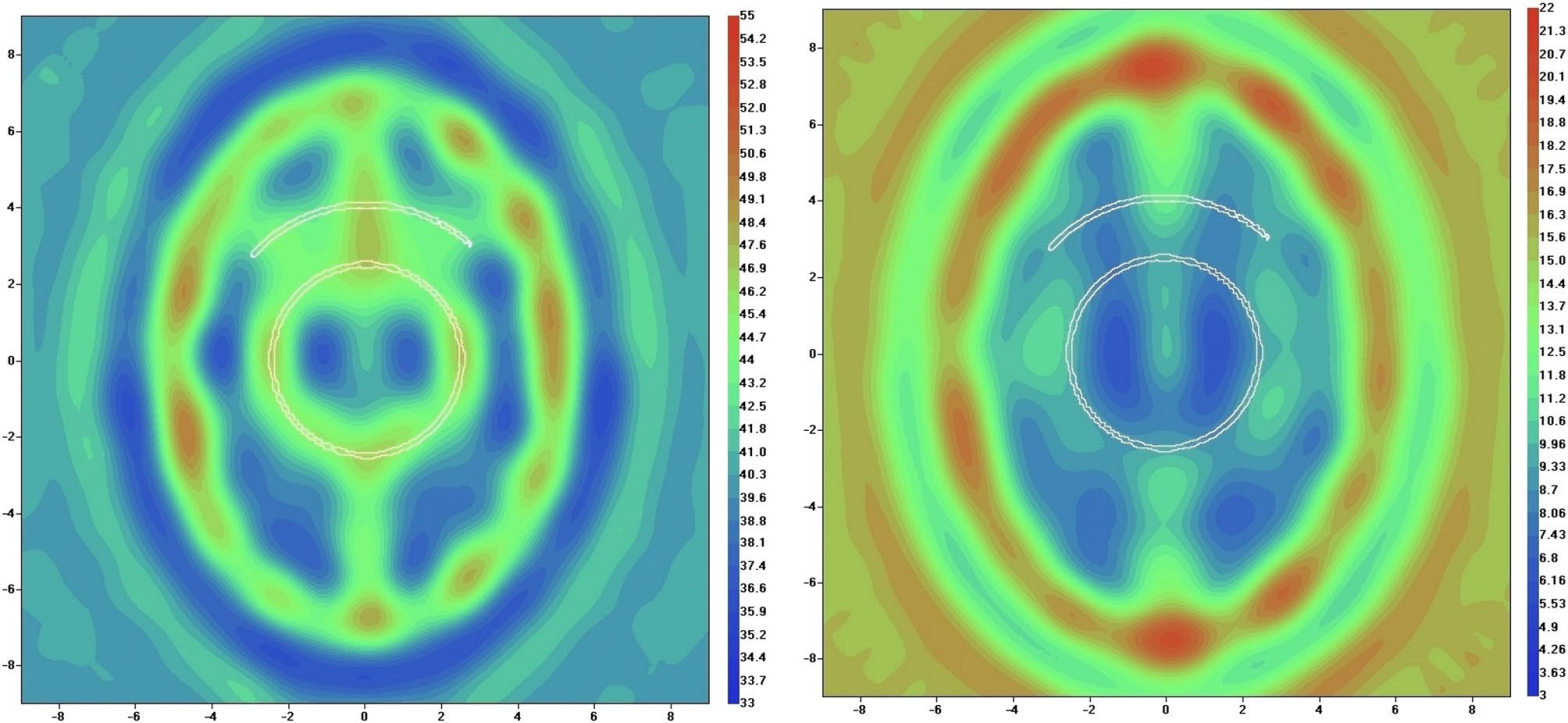
Reconstructed images of human head model 1 in systolic phase using EMT angiography approach: left is real part (at 50th iteration) and right is imaginary part (at 25th iteration) of dielectric properties. Scales are in [cm]. The counter of vessel is white coloured.

Reconstructed images of a human head model 1 in systolic phase are presented in Fig. 4. The iteration process has converged to presented images within 50 iterations with high convergence rate of 0.99. The images show skull and inner structure of the brain, but no vasculature has been reconstructed. Reconstructed images of a human head model 1 in systolic phase using EMT angiography approach are presented in Fig. 5. The convergence rate was 0.96 at 25th iteration and growth slightly to 0.99 at 50th iteration. The vasculature is clearly visible especially in the real part of the reconstructed images. The parameter α (Eq. (2)) was chosen by a trail method and set at the constant value of 100 during the whole iteration process.

**Figure 6:**
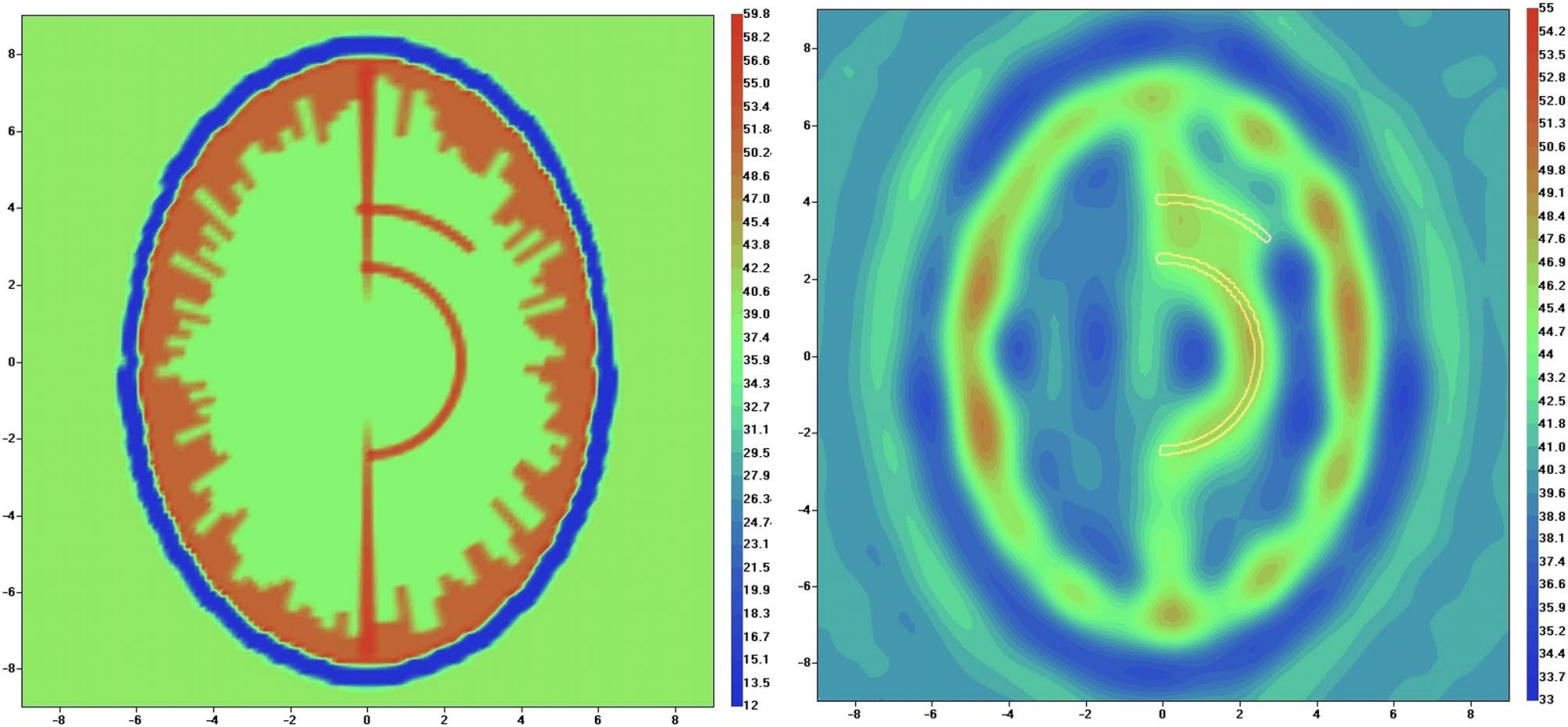
Virtual model of human head with circulation blockage in left hemisphere (left, real part of dielectric properties) and reconstructed image using EMT angiography approach (right, real part of dielectric properties). Scales are in [cm]. The counter of vessel is white coloured.

The virtual model of human head (model 1) with circulation blockage in left hemisphere (left, real part of dielectric properties) and reconstructed image using EMT angiography approach (right, real part of dielectric properties) are presented in Fig. 6. The circulation blockage in left hemisphere is clearly identified in reconstructed image with convergence rate of 0.99. Similar as above, the parameter α (Eq. (2)) was chosen by a trail method and set at the constant value of 100 during the whole iteration process. The reconstruction using standard EMT method fails to reconstruct the vasculature.

**Figure 7:**
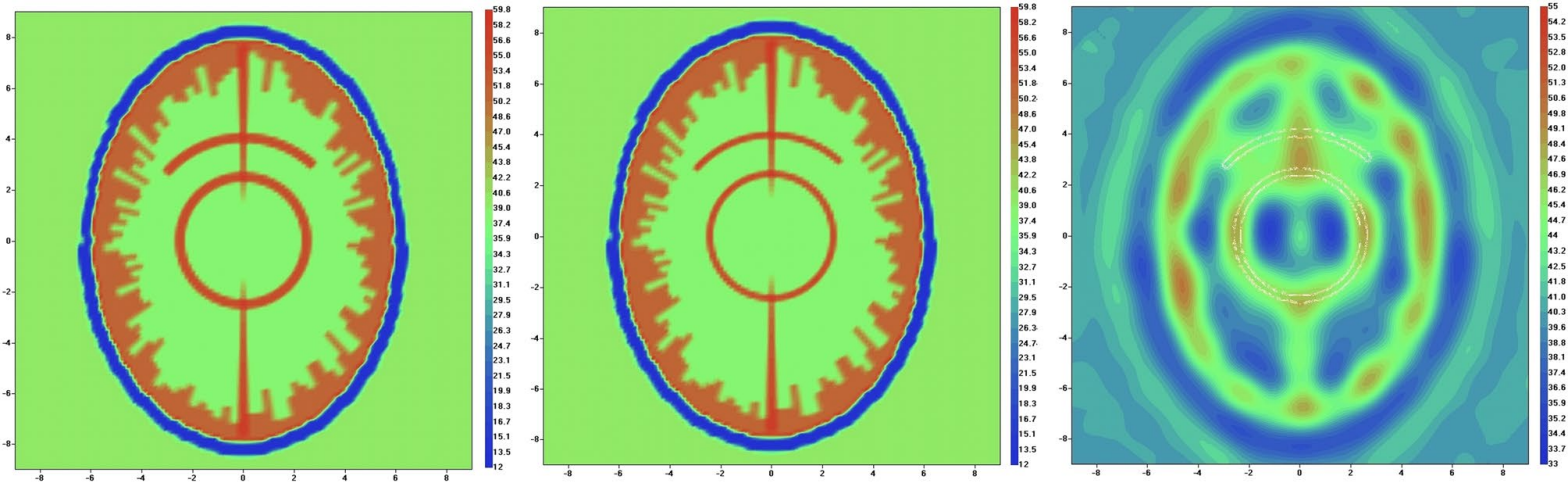
Virtual mode of human head (model 2) and reconstructed image. The systolic phase model is shown on the left-hand panel and diastolic phase model is shown on the mid-hand panel for real part of the dielectric properties. The reconstructed image is shown in right-hand panel for real part of the dielectric properties. Scales are in [cm]. The counter of vessel is white coloured.

The virtual model of human head (model 2) at systolic phase (left) and at diastolic phase (center) is shown on Fig. 7. The reconstructed image using EMT angiography approach is presented in Fig. 7 (right). The convergence rate during 50 employed iterations was 0.98. The vasculature is clearly visible. The parameter α (Eq. (2)) was chosen by a trail method and set at the constant value of 200 during the whole iteration process. The reconstruction using standard EMT method fails to reconstruct the vasculature.

**Figure 8:**
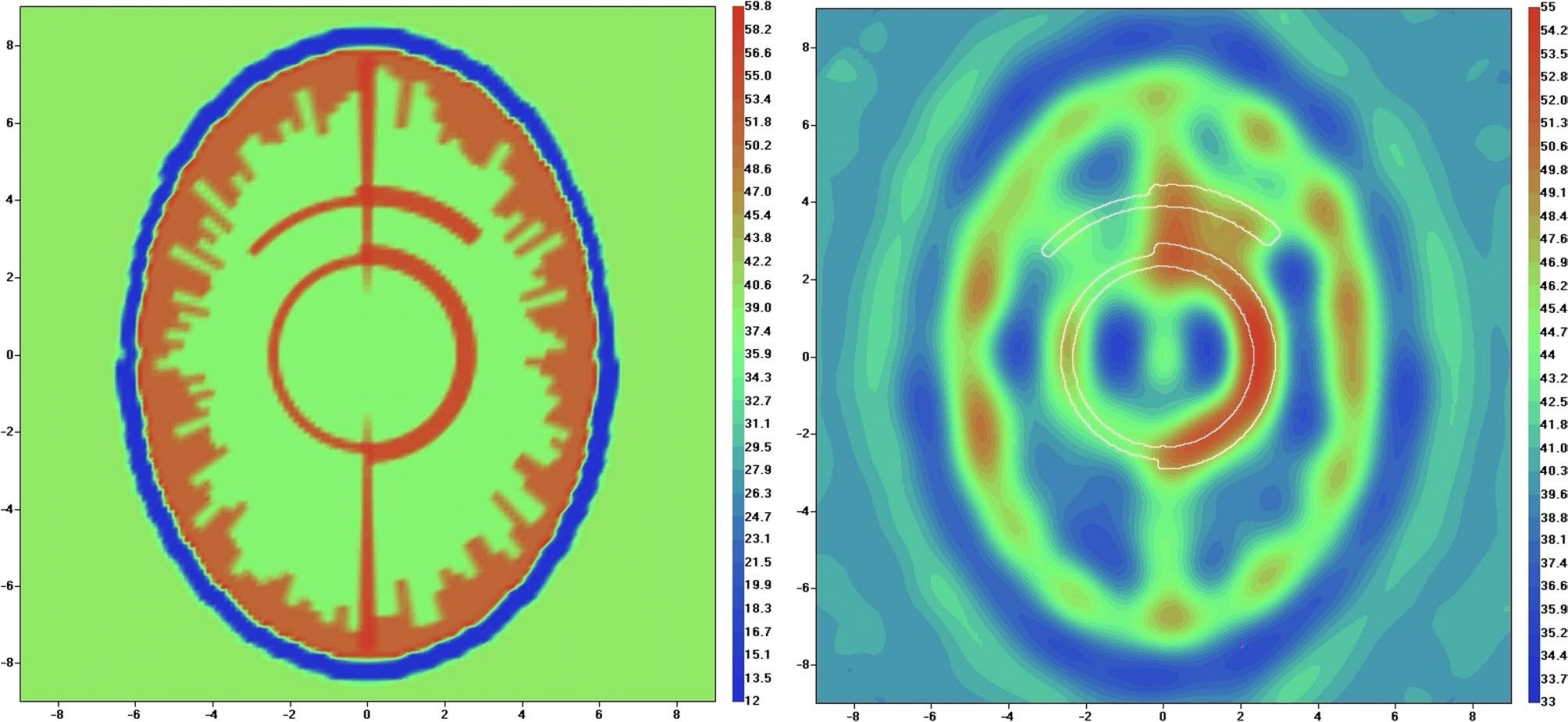
Virtual mode of human head with different dimensions of the vessels (model 1, systolic phase, left) and reconstructed image (right). Scales are in [cm]. The counter of vessel is white coloured.

Next, we modified model 1 in such a way that the model incorporates differently sized vasculature. Particularly, we keep XY dimensions of the vessels at 1.3 mm in right semi-sphere, while increased it to 3.9 mm in left semi-sphere (Fig. 8, left, real part of dielectric properties). The reconstruction using standard EMT method fails to reconstruct the vasculature. The reconstruction image using EMT angiography method (real part) is shown on Fig. 8 for 50th iteration with convergence rate of 0.98. Different dimensions of the vessels are clearly identified on the reconstructed image (Fig. 8, right, real part of dielectric properties). The parameter α (Eq. (2)) was chosen by a trail method and set at the constant value of 100 during the whole iteration process. It is understood that the issue of “calibration” of the suggested method in defining exact dimensions of vessels remains open and will be an issue of future in-depth research. With this study two hypothesis have been proven:

1. EMT angiography is capable for detecting small blood vessels within mm dimensions.

2. EMT angiography is sensitive to variations in dimensions of blood vessels.

There are also important scientific questions remain to be experimentally answered, specifically, what we are sensing with the EMT angiography—blood volume or oxyHb/deoxyHb ratio or combinations?

### Technical requirements for angio-capable EMT system

#### Frequency

When choosing a probing frequency or a band of multi-frequencies for EMT one should consider few controversial factors. With obvious desire of having higher detectability (resolution) the trend is to move to higher frequencies. However, this motion is restricted by high attenuation of EM radiation within biological tissues and the desire to operate at a short data-acquisition time, ideally within a fraction of typical human circulation time (tens to hundred [msec]). A “functional” sensing capability should be also taken into account: at high microwave frequencies the predominant sensing factor is water (either free or bound water), while at lower GHz to hundreds of MHz portion of EM spectrum, EM radiation senses functionally important cellular/membrane composition and ions’ conductivity of tissue in addition to water. This is evidenced by frequency dependences of experimentally observed functional responses to, for example, tissue hypoxia and blood flow reduction^8,9^. Prof Herman Schwan estimated that the conductivity contribution in tissue with high (75%) water content at frequency band near 1 GHz is the following: ions—80%; water (free and bound)—18% and proteins—2%, while at frequency near 0.5 GHz it is about 92%, 6% and 2% correspondingly^32^. Weighting all above factors using prior experience in EMT application for human brain imaging, we estimate that an optimal frequency band for human brain EMT imaging is about 0.9 to 1.5 GHz. The upper limit can go higher if antennas are to be positioned directly on a human head or via a small portion of gel (similar to the approach used in ultrasound).

#### Attenuation

As noted above, human brain tissues shielded by a high dielectric contrast shield, comprising the skull with low dielectric permittivity and cerebrospinal fluid with high dielectric permittivity: from 12 to 60 correspondingly at GHz region of EM spectrum. The shield effectively reflects extramural EM radiation from affecting brain tissues. In order to image deep brain tissue, the use of so-called matching media is essential in classical EM tomography. The dielectric properties of such media are chosen to be closer to the ones of brain tissue, decreasing reflection of EM radiation. Therefore, an imaging domain of EM tomography consists of highly absorptive medias: a human head to be studies surrounded by a matching media with sensors (antennas) located at the outer boundary of an imaging domain. Within a cylindrical imaging domain of radius 14.5 cm filled with matching media of about ε = 45*j*20, when a human head is inside the domain the measured attenuations are about 113 dB at 1 GHz, based on the data from 19 patients (approved clinical study protocol, unpublished data). Together with the need of having good signal to noise (S/N) ratio of about 20-30 dB for proper image reconstruction, it leads to challenging requirements, taking into account the limitations by a short data acquisition time (hundred of msec max) for cardiac synchronization acquisition. The synchronization of EMT data acquisition with circulation (for example with R-wave of ECG) is must have feature of angiography capable EMT system.

#### Antennas and RF electronics

Antennas should have predictable radiation pattern, easy to be approximated by analytical function, for example dipole models. This will help to optimize the number of numerical grids and to speed-up solution of direct problem. From another side, for example when using a cylindrical imaging domain, the dimensions of antennas should be minimized in order to have at least 64 antennas on the cross-sectional perimeter of a domain (64 antennas on the ring) with at least five antennas’ rings. This will lead to a total of 320 antennas with 320 RF transmitting/receiving (TxRx) circuits. Each of these 320 TxRx units should be capable both to irradiate and to acquire complex, highly attenuated EM signals within a short data acquisition time (see above). To add additional challenge to already challenging technical problem, the one should consider the need of EM isolation in-between TxRx channels and the need of cooling down the complex electronic system with 320 TxRx units.

Giving the capability of EMT for a short data acquisition within [msec] range, there are potentials for a dynamic EMT angiography, meaning capability to look at vessels’ functionality in the dynamics of circulatory activity, ie at each cardiac cycle.

## Conclusions

New method of electromagnetic tomographic angiography was presented in application to cerebral angiography. Achieved imaging results clearly demonstrate applicability of the method for detecting small cerebral vessels of the diameter as small as 1.3 mm and distinguish vessels with different dimensions. It is understood that the issue of “calibration” of the suggested method in defining exact dimensions of vessels remains open and will be an issue of future in-depth research. With this study two hypothesis have been proven:

1. EMT angiography is capable for detecting small blood vessels within mm dimensions.

2. EMT angiography is sensitive to variations in dimensions of blood vessels.

The technical challenges in the development of angiography capable EMT systems are assessed and discussed.

## Data Availability

The datasets used and/or analysed during the current study available from the corresponding author on reasonable request.
